# Applications of Multiple Nuclear Genes to the Molecular Phylogeny, Population Genetics and Hybrid Identification in the Mangrove Genus *Rhizophora*

**DOI:** 10.1371/journal.pone.0145058

**Published:** 2015-12-16

**Authors:** Yongmei Chen, Yansong Hou, Zixiao Guo, Wenqing Wang, Cairong Zhong, Renchao Zhou, Suhua Shi

**Affiliations:** 1 State Key Laboratory of Biocontrol and Guangdong Provincial Key Laboratory of Plant Resources, School of Life Sciences, Sun Yat-sen University, Guangzhou, Guangdong, China; 2 School of Life Science, Xiamen University, Xiamen, Fujian, China; 3 Hainan Dongzhai Harbor National Nature Reserve, Haikou, Hainan, China; 4 School of Chemistry and Pharmaceutical Engineering, University of Sichuan Science and Engineering, Zigong, China; National Cheng-Kung University, TAIWAN

## Abstract

The genus *Rhizophora* is one of the most important components of mangrove forests. It is an ideal system for studying biogeography, molecular evolution, population genetics, hybridization and conservation genetics of mangroves. However, there are no sufficient molecular markers to address these topics. Here, we developed 77 pairs of nuclear gene primers, which showed successful PCR amplifications across all five *Rhizophora* species and sequencing in *R*. *apiculata*. Here, we present three tentative applications using a subset of the developed nuclear genes to (I) reconstruct the phylogeny, (II) examine the genetic structure and (III) identify natural hybridization in *Rhizophora*. Phylogenetic analyses support the hypothesis that *Rhizophora* had disappeared in the Atlantic-East Pacific (AEP) region and was re-colonized from the IWP region approximately 12.7 Mya. Population genetics analyses in four natural populations of *R*. *apiculata* in Hainan, China, revealed extremely low genetic diversity, strong population differentiation and extensive admixture, suggesting that the Pleistocene glaciations, particularly the last glacial maximum, greatly influenced the population dynamics of *R*. *apiculata* in Hainan. We also verified the hybrid status of a morphologically intermediate individual between *R*. *apiculata* and *R*. *stylosa* in Hainan. Based on the sequences of five nuclear genes and one chloroplast intergenic spacer, this individual is likely to be an F1 hybrid, with *R*. *stylosa* as its maternal parent. The nuclear gene markers developed in this study should be of great value for characterizing the hybridization and introgression patterns in other cases of this genus and testing the role of natural selection using population genomics approaches.

## Introduction

Mangroves are plants that inhabit the intertidal zones of tropical and subtropical coasts [1]. They consist of approximately 70 species from 28 genera in 20 families and provide important ecological services in coastal ecosystems [2]. The geographic distribution of mangroves can be divided into two main regions, the Indo-West Pacific (IWP) region and Atlantic-East Pacific (AEP) region [3]. Most mangrove species are distributed in the IWP region, and only 13 species occur in the AEP region [2]. This geographic anomaly of mangrove distribution has been attributed to different rates of origin of new mangrove lineages in the two regions after the initial origin around the Tethys Sea [4]. The majority of the mangrove genera are confined to either the IWP region or AEP region, with only three genera, *Rhizophora* L., *Avicennia* L. and *Acrostichum* L., common to both regions [2]. Why only some mangrove genera can disperse into two regions remains unclear.

*Rhizophora* (Rhizophoraceae) is one of the most common and widely distributed genera of mangroves [2–3]. This genus is composed of five species, among which three species, *R*. *apiculata*, *R*. *stylosa* and *R*. *mucronata*, are distributed throughout the IWP region and two species, *R*. *mangle* and *R*. *racemosa*, occur primarily in the AEP region [3]. What are the phylogenetic relationships between these species? When and where did these species originate? How were these species dispersed? Answering these questions can contribute to understanding the biogeographic history of mangroves. Previous phylogenetic analyses have demonstrated that the genus *Rhizophora* falls into two clades: the AEP clade and IWP clade [5–7]. However, these studies generated highly controversial results in terms of the estimation of the divergence time between the two clade, which causes the origin and dispersal histories of *Rhizophora* species to remain elusive [5–8].

Interspecific hybridization is common in this genus, with at least six hybrid taxa that have been reported [9–12]. Some hybrids of *Rhizophora* are sterile and limited to the F1 stage, such as *R*. × *lamarckii* and *R*. × *annamalayana* [10,12], whereas other combinations may produce advanced generation hybrids, such as the hybridization between *R*. *mucronata* and *R*. *stylosa* [11] and between *R*. *mangle* and *R*. *racemosa* [9], which implies different levels of reproductive isolation between different *Rhizophora* species. Additionally, introgression may occur between some species. There are two species of *Rhizophora* in Hainan, China, *R*. *apiculata* and *R*. *stylosa* [3]. *R*. *stylosa* is a dominant species in western and northern coasts of Hainan, but is relatively uncommon in eastern coasts of Hainan. *R*. *apiculata* is mainly distributed in the eastern coasts of Hainan from Sanya to Wenchang. Natural hybrids between the two species are relatively rare and have only been reported in some special localities of the IWP region [11–12]. To date, there have been no reports of natural hybridization between them in China. In 2013, we systematically investigated *Rhizophora* in Hainan and found an individual with morphological characteristics that are intermediate between *R*. *apiculata* and *R*. *stylosa* in the Yalong Bay-Qingmei Harbor Mangrove Nature Reserve, Sanya, Hainan. We propose that it may represent a hybrid between *R*. *apiculata* and *R*. *stylosa*. Because the morphological intermediacy is not always associated with hybridization [13], its hybrid status must be confirmed by molecular means.

Population genetics studies of mangroves can be used to infer their population history and assess their health status and evolutionary fate. Isozyme and PCR-based markers, such as random amplified polymorphic DNA (RAPD), inter-simple sequence repeat (ISSR), amplified fragment length polymorphism (AFLP) and simple sequence repeat (SSR) have been used in population genetics studies of mangroves in the recent past [14]. However, the number of markers used in these studies is often limited, and there are potential homoplasy issues for these anonymous bands. In addition, the reproducibility of the experiments and the transferability of these markers to other species are also not ideal. To our knowledge, there have been only five nuclear genes used for the genetic diversity and genetic structure analyses of three IWP *Rhizophora* species [15–16]. With viviparous propagules, species of *Rhizophora* are considered to have the potential for long-distance dispersal [17–18]. However, previous population genetics studies on species of *Rhizophora* revealed strong population differentiation, even between populations within a relatively small area [19–20]. This means that other evolutionary forces, such as demography and (or) natural selection, may also play important roles in the population structure of mangroves.

Nuclear gene markers are increasingly used in molecular phylogeny, population genetics and hybridization studies because the analysis of multiple independent nuclear loci across the genome can provide more comprehensive and precise information. In this study, we developed a set of nuclear exon-primed intron-crossing (EPIC) markers that are universal for all species of *Rhizophora*. We applied a small subset of these markers to reconstruct the molecular phylogeny of this genus, investigate the population genetics of *R*. *apiculata* and identify the hybrid status of *Rhizophora* in Hainan, China.

## Materials and Methods

### Plant materials and genomic DNA extraction

Leaf samples for each *Rhizophora* taxon were collected from their natural populations and dried using silica gels. Details regarding the sampling taxa, the geographical locations and the sample size used in this study are provided in Table 1. Two accessions of each species were used for phylogenetic analyses. Samples from four populations (QL, TL, YL and SY) in Hainan, China, were used for the population genetics study of *R*. *apiculata*. Samples from Yalong Bay, Hainan were used for the identification of hybridization. Genomic DNA was extracted from each individual using the CTAB method [21] with 100 mg of dried leaf tissues ground using a Tissuelyser II (QIAGEN, Hilden, Germany). The extracted genomic DNA was dissolved in 50 μL of 1× TE buffer and was stored at −20°C.

**Table 1:** Sampling information for the six *Rhizophora* taxa and the outgroup species (*Bruguiera gymnorrhiza*) used in this study.

Taxon	Sampling location (population code)	Geographical coordinates	Sample size
***R*. *apiculata***	Qinglan Harbor, Wenchang, Hainan, China (QL)	110.79°E, 19.61°N	30
	Tielu Harbor, Sanya, Hainan, China (TL)	109.70°E, 18.25°N	21
	Yulin River, Sanya, Hainan, China (YL)	109.59°E, 18.28°N	21
	Sanya River, Sanya, Hainan, China (SY)*	109.50°E, 18.24°N	30 (1)
	Ngao, Ranong, Thailand*	98.58°E, 9.87°N	1 (1)
	Yalong Bay, Sanya, Hainan, China	109.61°E, 18.24°N	10
**Putative hybrid**	Yalong Bay, Sanya, Hainan, China	109.61°E, 18.24°N	1
***R*. *stylosa***	Yalong Bay, Sanya, Hainan, China*	109.61°E, 18.24°N	12 (1)
	South Alligator River, Northern Territory, Australia*	132.38°E, 12.40°S	1 (1)
***R*. *mucronata***	Chaiya, Surat Thani, Thailand*	99.25° E, 9.37°N	1 (1)
	Mida Creek, Watamu, Kenya*	39.96° E, 3.38°S	1 (1)
***R*. *mangle***	Bodo estuarine, Rivers State, Nigeria*	7.26° E, 4.56°N	2 (2)
***R*. *racemosa***	Bodo estuarine, Rivers State, Nigeria*	7.24°E, 4.61°N	2 (2)
***B*. *gymnarrhiza***	Qinglan Harbor, Wenchang, Hainan, China*	110.79°E, 19.61°N	1 (1)

* in the second column indicates that one or two individuals of this population were used in the phylogenetic analyses. Numbers in parentheses in the fourth column are sample sizes used in the phylogenetic analyses.

### Primer design, PCR and sequencing

To develop new nuclear gene primers for all of the *Rhizophora* species, the transcriptome sequences of *R*. *mangle* were downloaded from the Mangrove Transcriptome Database (http://mangrove.illinois.edu/transcriptome). To anchor the conserved regions for the primer design, we also downloaded the expressed sequence tags (ESTs) of *Bruguiera gymnorrhiza* from NCBI [22] because *Bruguiera* is closely related to *Rhizophora* [23]. We performed the following steps for the primer design: (1) A Blastx search against the NCBI database was performed and the sequences with well-annotated coding regions and exon/intron boundaries were selected. (2) The sequences containing more than two exon regions were chosen and primer pairs that anchor on two different exons were designed using Primer Premier v.5.0 (Premier Biosoft International, Palo Alto, CA, USA). (3) Primer pairs with a rough PCR product length ranging from 500 to 1,500 bp were selected. We also used four known pairs of nuclear gene primers, *DLDH*, *mang*-1, *PAL1* and *SBE2* [15], and one pair of chloroplast primers, *trn*S-*trn*G [24], in this study. All of the primer sequences are listed in S1 Table. All of the primers were synthesized at Life Technologies Corporation (Shanghai, China).

The PCR amplifications were performed under the following conditions: 94°C for 4 min, 35 cycles at 94°C for 30 s, at the annealing temperature (Ta) for 30 s, and at 72°C for 1.5 min, followed by a final extension step at 72°C for 8 min. The Ta was adjusted based on the result of the PCR amplification. The PCR reaction was conducted in a reaction volume of 50 μL, with 50 ng of DNA, EasyTaq DNA Polymerase (Transgen Biotech, Beijing, China) and other components. The amplified products were detected electrophoretically on a 1% agarose gel and visualized under a UV light.

The PCR products that were used for the sequencing were purified via a 1.2% agarose gel electrophoresis followed by the use of a Pearl Gel Extraction Kit (Pearl Bio-tech, Guangzhou, China). The purified PCR products were sequenced on an ABI 3730 DNA automated sequencer at BGI, Shenzhen, China.

### Data analysis

The DNA sequences were assembled using SeqMan v.6.0 (DNASTAR, Madison, USA), were aligned using Clustal X [25] and were further edited by hand. The putative functions and coding regions of the nuclear genes were determined using Blastx from NCBI. The phylogenetic analyses using the maximum parsimony (MP) and maximum likelihood (ML) methods were performed using MEGA v.6.06 [26], and the divergence times were estimated using the Bayesian Markov chain Monte Carlo (MCMC) method in Beast v.1.8.1 [27]. The sequences with five nuclear loci used for the phylogenetic analyses were concatenated, and any gaps were deleted. The best nucleotide substitution model (Hasegawa-Kishino-Yano model) was determined using jmodeltest v.2.1.5 [28] with the Bayesian Information Criterion (BIC) method. In the phylogenetic analyses for the MP and ML methods, the number of bootstrap replications was 1,000. In the Beast analysis, the lognormal relaxed clock model and Yule process tree prior were applied, assuming independent rates on different branches and a constant speciation rate per lineage; two independent MCMC runs were performed for 50,000,000 generations and were sampled every 1,000 generations. The posterior probabilities and other parameters were shown to converge after 100,000 generations, and the first 500 trees out of 50,000 trees were discarded. Assuming that the common ancestor of *Rhizophora* and *Bruguiera* is older than the extensive fossil records of *Rhizophora* (~45 Mya) or *Bruguiera* (~50 Mya) but not older than the origin of the Rhizophoraceae family (~60 Mya) [8], we set the root node of the lognormal prior distribution with an offset value of 45, a mean of 2, and a standard deviation of 0.5 in the Beast analysis. Tracer v.1.6 [29] was used to verify the posterior distributions and effective sample size (ESS) of the parameters in the Beast analysis to ensure that the parameters converged and that the minimum ESS for each parameter was at least 2,000. FigTree v.1.4.2 [30] was used to draw the phylogenetic tree of the Beast analysis.

For the population genetics and hybridization analyses, the aligned sequences were phased using PHASE embedded in Dnasp v.5.0 [31]. The genetic diversity statistics in the four *R*. *apiculata* populations, including the sequence length, number of silent sites (including synonymous sites in the coding region and intron sites), number of sequences (n), number of segregating sites (S), number of haplotypes (h), haplotype diversity (Hd), nucleotide polymorphism per site (θw), nucleotide diversity per site (π_t_), nucleotide diversity per silent site (π_s_) and nucleotide diversity per nonsynonymous site (π_a_), were counted and calculated using Dnasp v.5.0 [31]. The haplotype distribution maps were drawn using GenGIS v.2.1.1 [32], and the haplotype networks were analyzed using the median joining algorithm in Network v.4.6.1.2 [33]. The AMOVA analysis, inbreeding coefficient (Fis), Hardy-Weinberg equilibrium tests, pairwise Fst and net average number of pairwise differences between the populations for each gene were calculated using Arlequin v.3.1 [34]. The pairwise Fst values of the seven nuclear genes were averaged, the standard deviations were calculated, and the net average numbers of pairwise differences between the populations for each gene were summed to represent the net genetic distance. We used the program STRUCTURE v.2.3.4 for the Bayesian clustering analysis [35]. We ran STRUCTURE using the admixture model as the ancestry model, and the allele frequencies correlated as the allele frequency model. The length of the burn-in period was 100,000, and the number of MCMC replications after the burn-in was 500,000. The K value was set from 1 to 10, and the number of independent runs for each K was 20. The mean L(K) and its deviation and delta K for each K were calculated using StructureHarvest v.0.6.94 [36]. The histograms of the frequencies of the genetic components for each individual were drawn using Distruct v.1.1 [37]. To estimate the population genetic parameters, including the effective population size, divergence time and migration rate, IM [38] was used to fit the isolation-with-migration model using an MCMC method for each population pair. The mutation rate of these genes for the IM analysis was based on the estimated mutation rate from the Beast analysis (8.90×10^−10^ (95% HPD: 8.07×10^−10^–9.79×10^−10^) per site per year), and the nucleotide substitution model was the HKY model. A generation time of twenty years was used in the IM analysis. The range of prior distributions were assessed and adjusted using several trial runs, and then, we performed two long independent runs with identical prior distributions and different random seeds. We used 1,000,000 burn-in steps and recorded the results every hour. We allowed the IM program to run for more than 11,447,254 steps to ensure that the lowest ESS values for each parameter were at least 500. When the two independent runs showed similar results, the run with higher ESS values was used for further analyses. The tails of the posterior distributions for all of the parameters should be complete or falling, and the smoothed highest posterior density values (HiSmth) for each parameter were used as the estimated values. As the three populations in Sanya are very close to each other, we also treated them as one population for estimating population genetics parameters.

### Ethics Statement

No endangered or protected species are involved at the sampling sites of this study and no specific permissions are required for the scientific research. The observational and field studies in Hainan, China were permitted and assisted by Hainan Dongzhai Harbor National Nature Reserve.

## Results

### Development of 77 nuclear gene markers in *Rhizophora*

We first designed approximately 200 pairs of EPIC primers based on the transcriptome sequences of *R*. *mangle* and the ESTs of *B*. *gymnorrhiza* [22,39]. Then, genomic DNA from *R*. *apiculata* was used to perform PCR amplifications for each primer pair, and the PCR products with bright bands were purified and sequenced. Finally, we obtained 77 pairs of primers that showed successful PCR amplification and sequencing in *R*. *apiculata*. We further tested the transferability of these markers in four other species of *Rhizophora*, *R*. *stylosa*, *R*. *mucronata*, *R*. *mangle* and *R*. *racemosa*. Our results showed that all 77 nuclear gene markers were transferable in the four other species of *Rhizophora*. The high transferability suggests relatively low sequence divergence between species of *Rhizophora*. We further tested four other previously reported nuclear gene markers, *DLDH*, *mang*-1, *PAL1* and *SBE2* [15], and again, they were all transferable in species of *Rhizophora*. Therefore, at least 81 universal nuclear gene markers for *Rhizophora* species are available. The primer sequences, annealing temperatures (Ta), sequence lengths for *R*. *apiculata*, putative functions and GenBank accession numbers are shown in S1 Table. We further tested the applications of these nuclear gene markers to the molecular phylogeny, population genetics and hybridization studies in the genus *Rhizophora*.

### Application I- Molecular phylogeny of *Rhizophora* and the divergence time estimation

**Fig 1:**
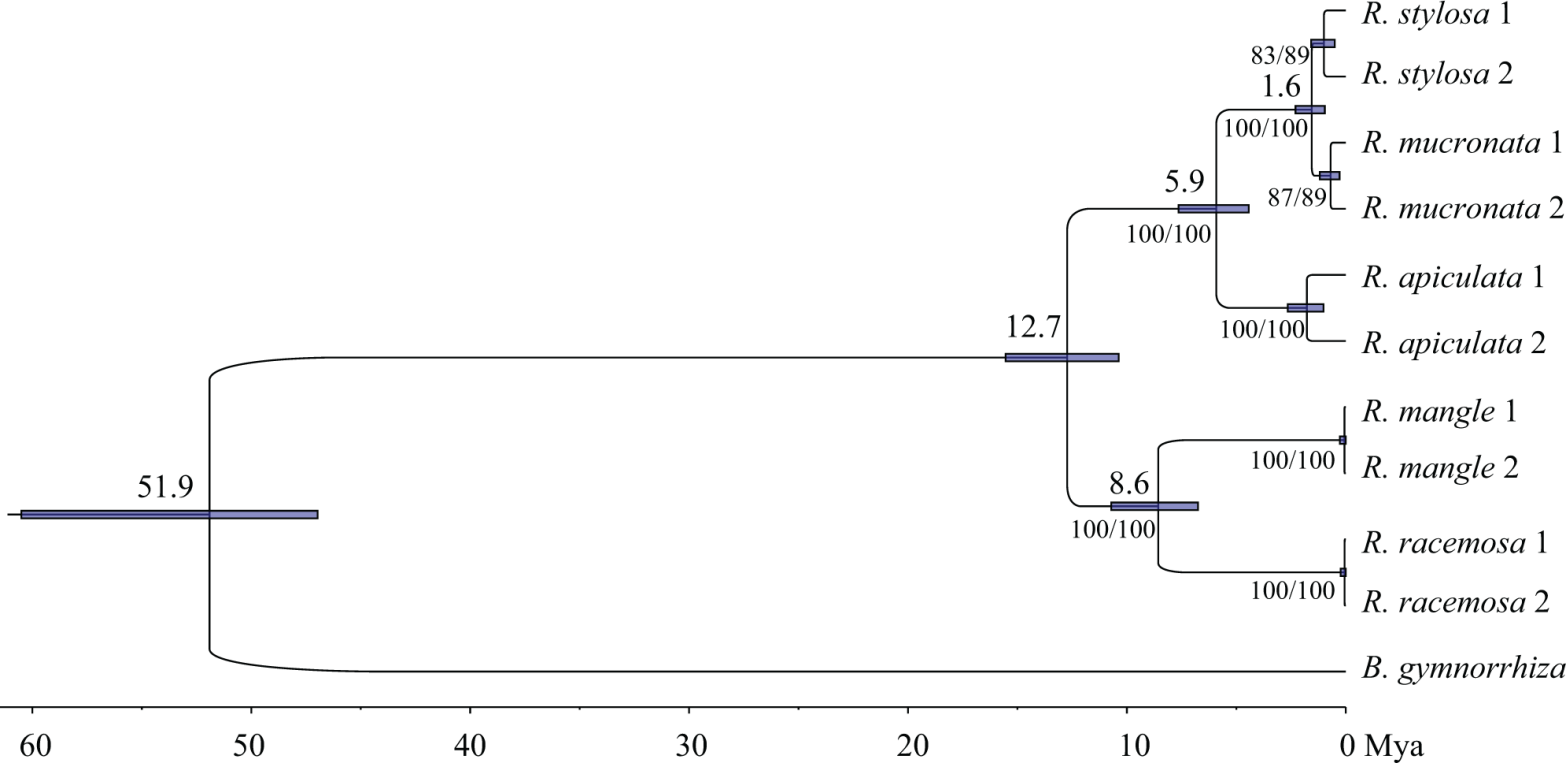
Phylogenetic tree of *Rhizophora* and the divergence time at each node of the tree. The phylogenetic tree and estimated divergence times are based on the Beast analyses. The numbers above the nodes represent the estimated divergence times (within-species divergence times are not shown), and the error bars represent the 95% HPD intervals of the estimated divergence times. The numbers below the nodes show the bootstrap supports of the maximum parsimony tree and the maximum likelihood tree using MEGA.

Previous phylogenetic studies have generated controversial results with regard to the origin and dispersal histories of *Rhizophora* species [5–8]. We first applied a part of the nuclear gene markers to reconstruct the phylogenetic tree and estimate the divergence time of the *Rhizophora* species. We sequenced five nuclear genes (*22454*, *23056*, *22274*, *23714* and *C49*) in all five *Rhizophora* species and in the outgroup species *B*. *gymnorrhiza*. These nuclear genes were randomly chosen as long as they contain intron(s) to ensure sufficient variation. For each of *R*. *apiculata*, *R*. *stylosa* and *R*. *mucronata*, two individuals were used for phylogenetic analyses and they were sampled from two geographically distant regions. For two other species in the AEP region, we had samples from only one location. Sequences of the five nuclear genes were concatenated and used for phylogenetic reconstruction using the Bayesian MCMC, maximum parsimony (MP) and maximum likelihood (ML) methods. All of the phylogenetic trees that were generated using these methods showed the same topology as previous studies (here, we only provide the phylogenetic tree that was generated using the Bayesian MCMC method). As shown in Fig 1, the genus *Rhizophora* fell into two clades: the AEP clade (*R*. *mangle* and *R*. *racemosa*) and the IWP clade (*R*. *apiculata*, *R*. *stylosa* and *R*. *mucronata*). Within the IWP clade, *R*. *apiculata* diverged first, and *R*. *stylosa* and *R*. *mucronata* are sister species. Two independent runs of the Beast analysis yielded highly similar results. The posterior probability for each node of the Bayesian MCMC tree was 1.00, and the bootstrap support of the MP and ML trees for each clade was at least 83% (Fig 1). The Bayesian MCMC analysis showed that the divergence time between the IWP and AEP clades of *Rhizophora* was 12.7 Mya (95% HPD, 10.4Mya-15.5 Mya). Within the IWP clade, *R*. *stylosa* and *R*. *mucronata* diverged 1.6 Mya (95% HPD, 0.96 Mya-2.3 Mya), whereas they diverged from *R*. *apiculata* 5.9 Mya (95% HPD, 4.4 Mya-7.6 Mya). The divergence time between the two *Rhizophora* species within the AEP clade was 8.6 Mya (95% HPD, 6.8 Mya-10.7 Mya).

### Application II- Population genetics study of *R*. *apiculata* in Hainan, China

Hainan is at the northern range margin of *R*. *apiculata*. *R*. *apiculata* is a dominate species in the eastern coast of Hainan Island from Sanya to Wenchang. The populations found at range margins are of great importance because they may harbor local adaptation [40–41], and further expansion will initiate from these populations in the context of global warming. We sequenced seven nuclear genes, *C22*, *22274*, *23056*, *23852*, *22*, *23186* and *22066*, in four natural populations of *R*. *apiculata* in Hainan, China. Again, these seven nuclear genes were randomly chosen as long as they contain the intron to ensure sufficient variation.

Extremely low levels of genetic diversity at all seven genes were observed in the *R*. *apiculata* populations in Hainan (Table 2). The nucleotide polymorphisms (θw) at the population level ranged from 0 to 1.59×10^−3^, the nucleotide diversity (π_t_) ranged from 0 to 2.54×10^−3^, the total θw at the species level ranged from 2.2×10^−4^ to 1.25×10^−3^, and the total π_t_ ranged from 6.5×10^−4^ to 2.2×10^−3^ (Table 2).

**Table 2:** Genetic diversity in the four *R*. *apiculata* populations in Hainan.

Locus	Analyzed length/Number of silent sites(bp)	Population	n	S	h	Hd	θw×10^3^	π_t_×10^3^	π_s_×10^3^	π_a_×10^3^
***C22***	658/440.5	QL	60	1	2	0.51	0.33	0.77	1.15	0.00
		TL	42	2	3	0.54	0.71	1.19	1.79	0.00
		YL	42	2	3	0.57	0.71	0.98	1.47	0.00
		SY	60	2	2	0.31	0.65	0.93	1.38	0.00
		Total	204	2	3	0.64	0.52	1.35	2.02	0.00
***22274***	1533/1322.0	QL	60	2	2	0.41	0.28	0.54	0.62	0.00
		TL	42	2	2	0.46	0.30	0.59	0.69	0.00
		YL	42	2	2	0.44	0.30	0.57	0.66	0.00
		SY	60	2	2	0.28	0.28	0.37	0.43	0.00
		Total	204	2	2	0.50	0.22	0.65	0.76	0.00
***23056***	441/330.8	QL	60	2	2	0.45	0.97	2.05	2.73	0.00
		TL	42	2	2	0.14	1.05	0.62	0.82	0.00
		YL	42	2	2	0.29	1.05	1.29	1.72	0.00
		SY	60	2	2	0.49	0.97	2.24	2.99	0.00
		Total	204	2	2	0.49	0.77	2.20	2.93	0.00
***23852***	1629/1332.8	QL	60	12	2	0.35	1.59	2.5	3.11	0.00
		TL	42	0	0	0.00	0.00	0.00	0.00	0.00
		YL	42	0	0	0.00	0.00	0.00	0.00	0.00
		SY	60	0	0	0.00	0.00	0.00	0.00	0.00
		Total	204	12	2	0.12	1.25	0.88	1.08	0.00
***22***	791/482.7	QL	12	2	2	0.41	0.84	1.03	1.70	0.00
		TL	12	2	2	0.30	0.84	0.77	1.26	0.00
		YL	12	2	2	0.17	0.84	0.42	0.69	0.00
		SY	60	2	2	0.26	0.54	0.66	1.07	0.00
		Total	96	2	2	0.27	0.49	0.67	1.10	0.00
***23186***	873/717.2	QL	12	3	2	0.53	1.14	1.82	2.22	0.00
		TL	12	3	3	0.44	1.14	0.85	1.04	0.00
		YL	12	1	2	0.17	0.38	0.19	0.23	0.00
		SY	58	1	2	0.37	0.25	0.43	0.52	0.00
		Total	94	3	3	0.53	0.67	0.79	0.96	0.00
***22066***	1418/1143.2	QL	12	2	2	0.53	0.47	0.75	0.93	0.00
		TL	12	3	3	0.32	0.70	0.45	0.56	0.00
		YL	12	2	2	0.53	0.47	0.75	0.93	0.00
		SY	12	1	2	0.30	0.23	0.21	0.27	0.00
		Total	48	4	4	0.66	0.64	0.94	1.17	0.00
**Total**	7343/5769.2		-	27	18	3.21	4.56	7.82	10.02	0.00
**Average**	1053.4/824.2		151	3.86	2.57	0.46	0.65	1.12	1.43	0.00

n: number of sequences; S: number of segregating sites; h: number of haplotypes; Hd: haplotype diversity; θw: nucleotide polymorphism per site. π_t_: nucleotide diversity per site. π_s_: nucleotide diversity per silent site, including the synonymous sites in the coding region and intron sites; π_a_: nucleotide diversity per nonsynonymous site.

A strong genetic structure was observed among the *R*. *apiculata* populations in Hainan based on the AMOVA analysis and pairwise Fst analysis. The AMOVA analysis showed that the overall Fst (from 0.187 to 0.492) for six of the seven genes was extremely significant, indicating strong genetic differentiation among the populations (Table 3). The average Fis of the QL, TL and YL populations were 0.199, 0.250, and 0.098, respectively, and the majority of the genes did not deviate significantly from Hardy-Weinberg equilibrium in these three populations (Table 3). However, the average Fis of the SY population was 0.631, which was considerably higher than the other populations, and three of the six polymorphic genes deviated significantly from Hardy-Weinberg equilibrium, indicating population admixture in SY population considering the subsequent results of the Bayesian clustering analysis. In general, the pairwise Fsts between the SY, TL and YL populations were lower, suggesting relatively recent divergence or more gene flow among the southern populations (Table 4).

**Table 3:** Overall Fst of the AMOVA analysis and the Fis inbreeding coefficient for each population at each nuclear locus.

Locus	Fst	Fis of QL	Fis of TL	Fis of YL	Fis of SY
***C22***	0.362 (***)	0.016 (ns)	-0.030 (ns)	-0.107 (ns)	0.237 (ns)
***22274***	0.272 (***)	0.277 (ns)	-0.263 (ns)	0.462 (*)	1.000 (***)
***23056***	0.300 (***)	0.117 (ns)	-0.053 (ns)	0.167 (ns)	0.667 (***)
***23852***	0.187 (***)	0.328 (ns)	-	-	-
***22***	-0.072 (ns)	0.615 (ns)	1.000 (ns)	0.000 (ns)	0.618 (**)
***23186***	0.304 (***)	0.706 (ns)	0.348 (ns)	0.000 (ns)	0.263 (ns)
***22066***	0.492 (***)	-0.667 (ns)	0.500 (ns)	0.063 (ns)	1.000 (ns)
**Average**	0.264	0.199	0.250	0.098	0.631

The significance of the Fst and Hardy-Weinberg equilibrium tests is shown in the parentheses following the Fst values and Fis values, respectively (ns: P > 0.05; *: 0.01 < P < 0.05; **: 0.001 < P < 0.01; ***: P < 0.001).

**Table 4:** Average pairwise Fst and its standard deviation (below diagonal), and the net genetic distance between the populations based on the seven nuclear genes (above diagonal).

	QL	TL	YL	SY
**QL**	-	2.102	3.774	3.250
**TL**	0.188 ± 0.215	-	2.346	0.911
**YL**	0.321 ± 0.187	0.253 ± 0.294	-	1.100
**SY**	0.321 ± 0.259	0.133 ± 0.201	0.132 ± 0.190	-

**Fig 2:**
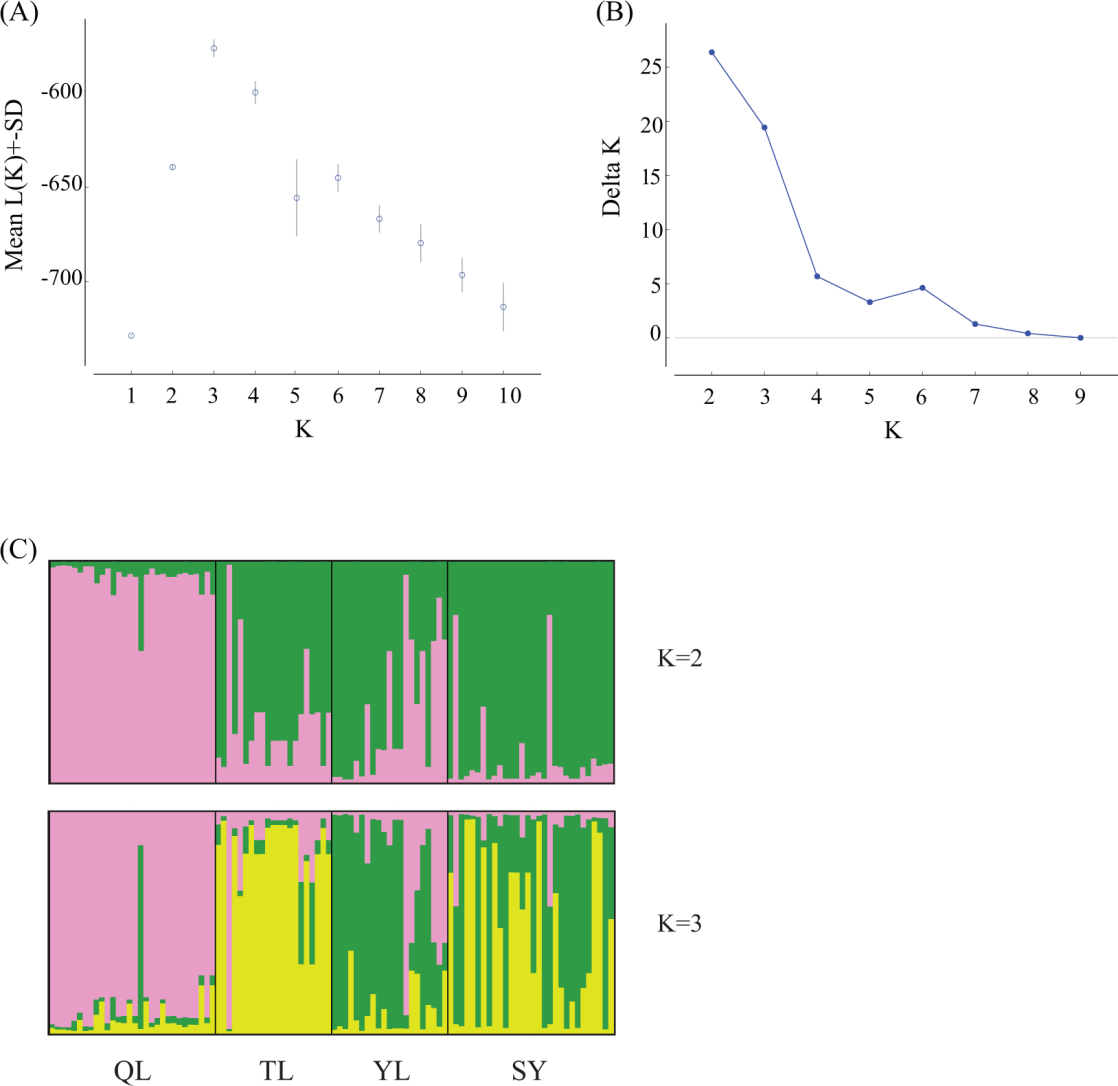
Results of the Bayesian clustering analysis. (A) The mean L(K) and its standard deviation for each K. (B) The delta K value for each K. (C) The histograms of the frequencies of the genetic components for each individual when K = 2 and K = 3.

The strong population differentiation was also supported by the Bayesian clustering analysis using STRUCTURE. The mean L(K) for each K reached the peak when K = 3 (Fig 2A); the delta K was largest when K = 2 and second largest when K = 3 (Fig 2B). The mean L(K) and delta K values suggested that these individuals could be grouped into two or three clusters. The frequencies of the genetic components of each individual when K = 2 and K = 3 are shown in Fig 2C. When K = 2, the QL population was distinct from the three southern populations. When K = 3, the QL, TL and YL populations were further separated, and the genetic composition of the individuals in the SY population was similar to either the TL cluster or YL cluster. The SY population appeared to experience a recent admixture with the TL and YL clusters.

Furthermore, we estimated the effective population sizes, divergence times and migration rates for these populations using the isolation-with-migration model, and the results are shown in Table 5. The effective population sizes of these populations ranged from 29.53 to 775.89, indicating small effective population sizes for these extant populations and the effective population sizes may be smaller considering the gene flow from nearby populations. However, the effective population sizes of the ancestral populations (N_A_) ranged from 19,199.17 to 32,176.05, suggesting population contraction in the past and (or) gene flow from distinct populations. The smaller migration rates were found between QL and other populations, whose population migration rate per generation (2N_1_m_1_ or 2N_2_m_2_) were all less than 0.08, indicating quite low levels of gene flow between the northern population and the southern populations of Hainan. The larger migration rates were found among the southern populations with the population migration rate per generation ranged from 0.00291 to 0.601, indicating higher, limited gene flow among the southern populations. This was consistent with the results of the pairwise Fst analysis and STRUCTURE analysis. We also treated the three southern populations as one population for estimating population genetics parameters, similar results were observed in this analysis (Table 5). The divergence times between the northern and southern populations ranged from 9,676 to 18,232 years ago, indicating the northern population was dispersed after the last glacial maximum.

**Table 5:** Estimated population genetics parameters of the *R*. *apiculata* populations in Hainan, China.

Population1	Population2	N_1_	N_2_	N_A_	t	2N_1_m_1_	2N_2_m_2_	m_1_	m_2_
**QL**	**TL**	275.48	29.53	32176.05	9676	4.67E-04	7.88E-02	8.47E-07	1.33E-03
**QL**	**YL**	310.75	118.58	30328.99	16226	1.58E-03	5.75E-02	2.54E-06	2.42E-04
**QL**	**SY**	222.75	34.32	32510.11	11151	3.78E-04	4.45E-02	8.47E-07	6.48E-04
**TL**	**YL**	775.89	103.84	22417.86	39683	1.87E-01	9.08E-02	1.21E-04	4.37E-04
**TL**	**SY**	486.88	92.69	21468.31	13744	4.49E-01	2.64E-01	4.61E-04	1.42E-03
**SY**	**YL**	312.42	225.09	19199.17	22455	2.91E-03	6.01E-01	4.65E-06	1.34E-03
**QL**	**TL+YL+SY**	530.67	163.70	30430.19	18232	9.00E-05	2.88E-01	8.47E-08	8.80E-04

The effective population sizes of population 1 (N_1_), population 2 (N_2_), and the ancestor population (N_A_), the population divergence time (t years), the population migration rate per generation (2N_1_m_1_) and the migration rate per generation (m_1_) from population 1 to population 2, and the population migration rate per generation (2N_2_m_2_) and the migration rate per generation (m_2_) from population 2 to population 1 were estimated using IM.

**Fig 3:**
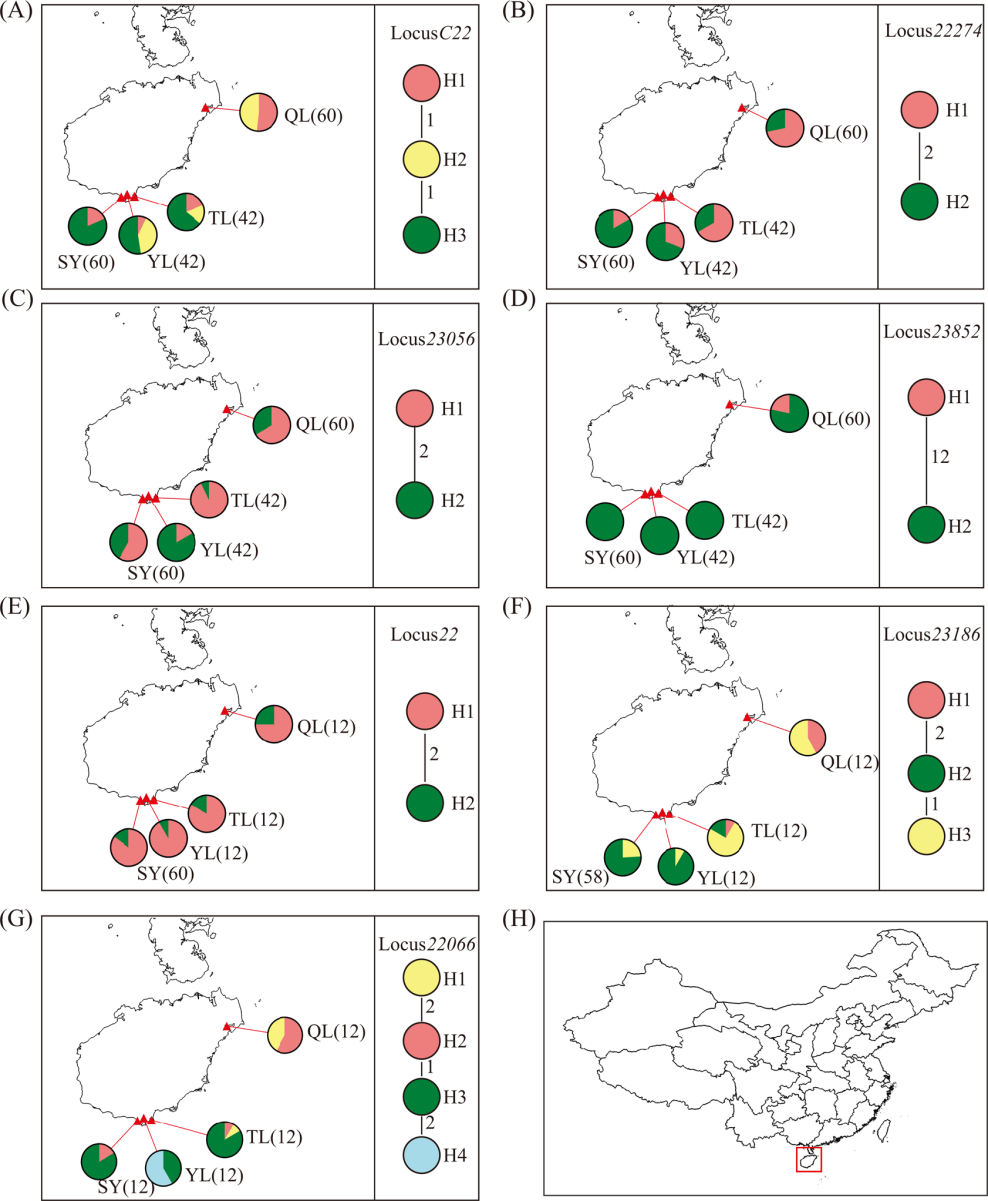
Maps of the haplotype distribution and haplotype networks of the seven nuclear loci. Figs A to G show the maps of the haplotype distribution and haplotype network for each locus. The red triangles on the map represent the sampling sites, and the black letters and numbers in the parentheses represent the population codes and the number of haplotypes, respectively. The frequencies of the haplotype components are shown using pie charts of the map. The circles with different colors in the haplotype networks represent different haplotypes, and the codes near the circles show the haplotype codes. The numbers in the connecting lines between the haplotypes represent the number of mutation steps. Fig H shows the location of Hainan in China.

The maps of the haplotype distribution and haplotype network for each nuclear gene are shown in Fig 3. The haplotype composition in the QL population differed markedly from the three other populations in Sanya at four loci, namely, *C22*, *23852*, *23186* and *22066*, suggesting that the northern population of QL was strongly differentiated from the southern populations at some loci. Notably, we found missing intermediate haplotypes at six of the seven loci in the haplotype networks, especially at locus *23852*, and there were no intermediate haplotypes found between two highly divergent haplotypes with 12 segregating sites. In the majority of the populations, we can see two divergent haplotypes at most or even all six loci, suggesting genetic admixture from two source populations.

### Application III- Identification of natural hybridization between *R*. *apiculata* and *R*. *stylosa* in Hainan, China

**Fig 4:**
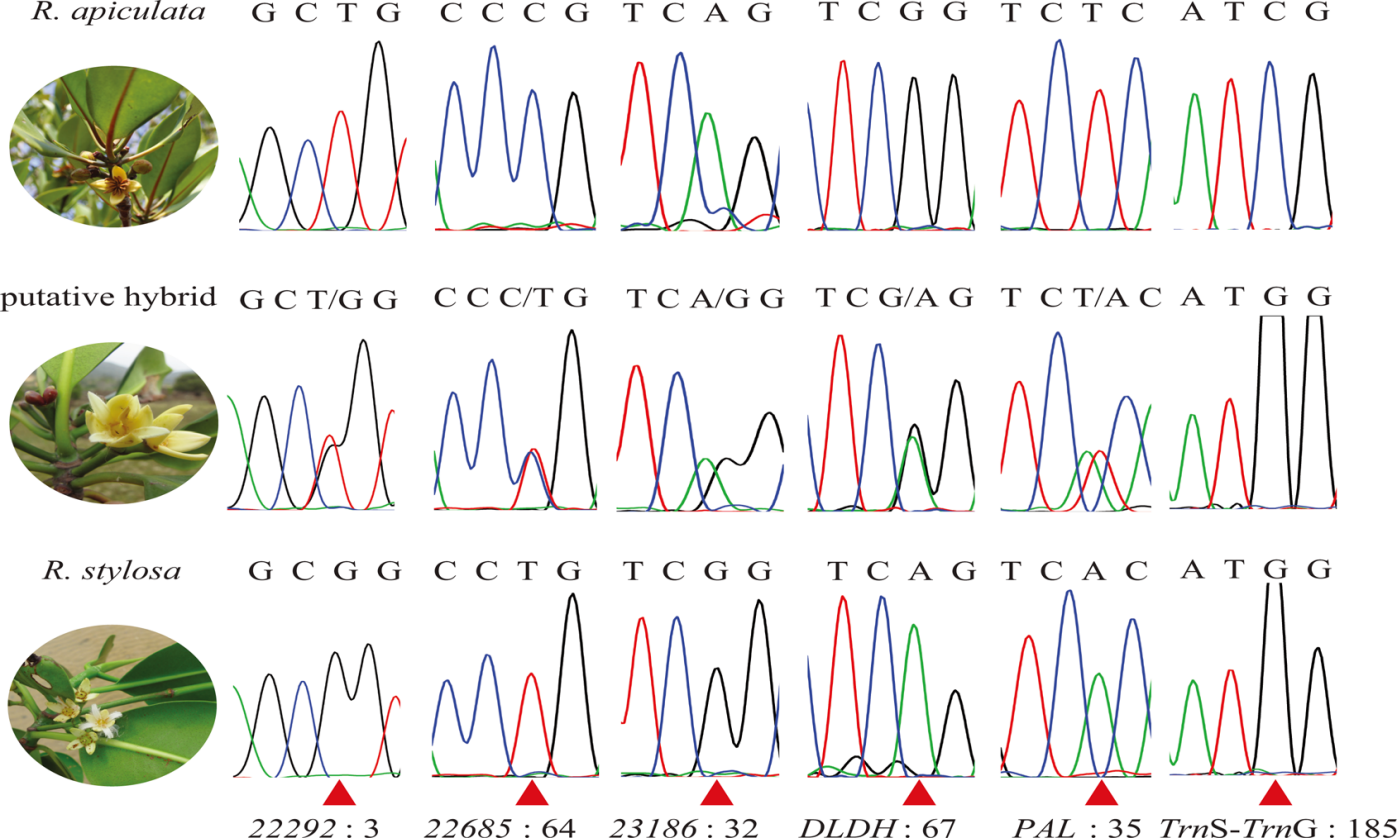
Chromatograms of the five nuclear genes and the chloroplast intergenic spacer in the three *Rhizophora* taxa in Hainan Island, China. Only one position for each locus is shown. The red triangles represent the variable sites between *R*. *stylosa* and *R*. *apiculata*, and the numbers after the colon represent the positions.

To verify the hybrid status of the morphologically intermediate individual in the Yalong Bay-Qingmei Harbor Mangrove Reserve, Hainan, China, we further sequenced five nuclear genes and one chloroplast intergenic spacer for the three *Rhizophora* taxa in this location. Ten and twelve individuals of *R*. *apiculata* and *R*. *stylosa*, respectively, were sequenced (Table 1). There was no sequence variation at any of the five nuclear genes among the ten *R*. *stylosa* individuals or at four of the five genes in the twelve *R*. *apiculata* individuals (S2–S6 Tables). *R*. *apiculata* had two haplotypes at the remaining gene (*22685*). In contrast, a substantial divergence between the two species was observed at these five genes, with 6 to 18 fixed mutations (including nucleotide substitutions and insertions/deletions) between them. Therefore, the two species are well separated. With regard to the putative hybrid individual, we observed sequence additivity in the chromatograms at all of the sites where *R*. *apiculata* and *R*. *stylosa* differed at each of the five genes (Fig 4; S2–S6 Tables). For gene *22685*, the sequence additivity was between *R*. *stylosa* and one haplotype (H2) of *R*. *apiculata* (S4 Table). Therefore, we provide compelling evidence for the hybrid status of this morphologically intermediate individual, and it is likely to be an F1 hybrid (*R*. × *lamarckii*). Chloroplast *trn*S-*trn*G sequencing showed that the sequence of this hybrid individual was identical to that of *R*. *stylosa* (Fig 4; S7 Table), indicating that *R*. *stylosa* is the maternal parent of this hybrid.

## Discussion and Conclusions

The genus *Rhizophora* is one of the most dominant and important components of mangrove forests in both the IWP and AEP regions. It is an ideal model system for studying biogeography, molecular evolution, population genetics, hybridization and conservation genetics of mangroves. However, as non-model plants, there are not sufficient molecular markers to deal with these questions. In this study, we developed 77 pairs of nuclear gene primers that showed successful PCR amplification in all five *Rhizophora* species and successful sequencing in *R*. *apiculata*. As a trial application, we used a subset of these markers to reconstruct the phylogeny, estimate the divergence times between nodes, examine the genetic structure and verify natural hybridization in this genus. We demonstrated the successful application of these markers and produced interesting results.

Previous studies have produced controversial conclusions regarding the divergence time between the IWP and AEP clades of *Rhizophora*. Based on the chloroplast and ITS data, the split between the two clades was estimated to have occurred approximately 47.6 Mya [7] or 11 Mya [5–6,8]. Based on our divergence time estimation, the IWP and AEP clades of *Rhizophora* began to diverge 12.7 Mya, which is slightly earlier than the results of Schwarzbach & Ricklefs [5–6], which were more recent than the worldwide fossil records of *Rhizophora* (~40 Mya [8]) and the closure of the Tethys Sea (~30 Mya [3]). Therefore, it is likely that the divergence between the extant IWP and AEP *Rhizophora* species was not caused by the closure of the Tethys Sea. Rather, our results support the hypothesis that *Rhizophora* first originated around Tethys Sea and later dispersed worldwide (~40 Mya), then began to disappear from the AEP region 14–15 Mya and was re-colonized into the AEP region from the IWP region approximately 12.7 Mya [5–6,8]. *Rhizophora* propagules have considerable longevity at sea [18], and propagules of *R*. *mangle* can survive over a year in seawater [17]. This long-distance dispersal ability makes it possible for the re-colonization of *Rhizophora* into the AEP region. In addition, the divergence between the two AEP *Rhizophora* species *R*. *mangle* and *R*. *racemosa* estimated in this study is considerably larger than those estimated by Schwarzbach & Ricklefs [5–6] and Lo et al. [7]. Hybridization and introgression have been reported between the two AEP *Rhizophora* species [9], and differential gene flow between the two species in different locations may have caused the inconsistency in the estimated sequence divergence. Because the majority of the data for the divergence time estimation in previous studies [5–7] were chloroplast DNA data, the chloroplast capture due to the hybridization may have caused an underestimation of the divergence. Our estimation using multiple nuclear genes is expected to be more accurate.

The extremely low genetic diversity, strong population differentiation and extensive admixture in populations of *R*. *apiculata* at its northern range margin were likely caused by the Pleistocene glaciations, particularly the last glaciation. In the Pleistocene period, the sea levels fluctuated dramatically and repeatedly, which strongly influenced the coastal mangrove forests [42–44]. For example, during the last glacial cycle, the sea levels fell from +6 m 120 Kya to approximately -120 m during the last glacial maximum (LGM; 19–26 Kya) [44]. In the LGM, the coastal areas of Hainan disappeared with the emergence of the Sunda Shelf in Southeast Asia, and the mangrove forests were mainly restricted to a narrow area on the outer margins of the shelf [43–44]. The estimated divergence times among the Hainan populations were all during the last glacial cycle and were mainly during the post-LGM period. Therefore, the extant mangrove forests in Hainan were likely established via post-glacial colonization from refugia. Population contractions during glaciations and (or) founder effect during the re-colonization process could have led to the low genetic diversity of the *R*. *apiculata* populations in Hainan, and genetic drift in small populations can cause strong genetic differentiation. If the re-colonization was from multiple source populations, admixture is also expected. A recent study showed that populations of *R*. *apiculata* in Southeast Asia were divided into two highly differentiated clusters with the Malay Peninsula as the boundary [16]. The missing intermediate haplotypes and two highly divergent haplotypes found in *R*. *apiculata* populations in Hainan may be caused by admixture of the two clusters. Low genetic diversity and strong population differentiation may also occur in marginal populations that are strongly shaped by local adaptation [39,41]. Additionally, the SY population is located in the city center of Sanya, and the natural mangrove forests have decreased rapidly and became fragmented during the past 50 years due to human disturbance. This would have reduced the gene flow within the population, which may have caused the higher inbreeding rate in the SY population. The recent admixture in the SY population may have occurred during the restoration of this mangrove forest.

Natural hybridization between *R*. *apiculata* and *R*. *stylosa* reported in this study is in the northernmost range margin of *R*. *apiculata*. The abundance of *R*. *stylosa* is far less than that of *R*. *apiculata* in Yalong Bay-Qingmei Harbor, and this is likely to be the reason why *R*. *stylosa* receives pollens from *R*. *apiculata* [45]. Multiple nuclear markers make the inference of the extent of hybridization feasible. In our case, it should be an F1 hybrid based on its heterozygous state for all of the investigated loci. Most cases of natural hybridization in mangroves appear to be restricted to the F1 generation [1,12,46–48], implying strong postzygotic isolation between congeneric species in mangroves. Only one hybrid individual was found in this case, indicating the hybridization between the two species was rare in Hainan. Because each of the parental species is more similar to the F1 hybrid than to each other, it may be easier for hybridization between F1 hybrid and their parental species. Thus the possibility of backcrossing and introgression still remains, although hybridization between *R*. *apiculata* and *R*. *stylosa* is detected rare. Gene flow between species can transfer adaptive traits and cause rapid adaptation [49–51]. The evidence for introgression in *Rhizophora* has been reported in two cases, between *R*. *stylosa* and *R*. *mucronata* and between *R*. *mangle* and *R*. *racemosa* [9,11,52]. However, whether the introgression, at least at some loci, is adaptive or not remains unclear. Moreover, some putative hybrid taxa in *Rhizophora*, such as *R*. *samoensis* var. *neocaledonica* and *R*. × *tomlinsonii* [10], have not been affirmed by molecular means, and the extent of hybridization remains unclear. With the large number of nuclear markers that were developed in this study, we can investigate more comprehensive patterns of hybridization and introgression in the genus *Rhizophora* and the underlying evolutionary forces.

## Supporting Information

S1 Table Primer sequences, annealing temperatures (Ta), sequence lengths (for *R*. *apiculata*) and putative functions of 81 nuclear gene markers for the mangrove genus *Rhizophora*. (XLS)

S2 Table The variable sites at the nuclear gene *22292* in three taxa of *Rhizophora* in Hainan. (XLSX)

S3 Table The variable sites at the nuclear gene *23186* in three taxa of *Rhizophora* in Hainan. (XLSX)

S4 Table The variable sites at the nuclear gene *22685* in three taxa of *Rhizophora* in Hainan. (XLSX)

S5 Table The variable sites at the nuclear gene *DLDH* in three taxa of *Rhizophora* in Hainan. (XLSX)

S6 Table The variable sites at the nuclear gene *PAL1* in three taxa of *Rhizophora* in Hainan. (XLSX)

S7 Table The variable sites at the chloroplast gene *trn*S-*trn*G in three taxa of *Rhizophora* in Hainan. (XLSX)
